# Associations between dairy farm performance indicators and culling rates under policy-driven herd size constraints

**DOI:** 10.3389/fvets.2023.1062891

**Published:** 2023-03-23

**Authors:** Pranav Shrikant Kulkarni, Monique Mourits, Mirjam Nielen, Wilma Steeneveld

**Affiliations:** ^1^Business Economics, Social Sciences Group, Wageningen University and Research, Wageningen, Netherlands; ^2^Department of Population Health Sciences, Farm Animal Health, Faculty of Veterinary Medicine, Utrecht University, Utrecht, Netherlands

**Keywords:** longevity, replacement, phosphate, herd characteristics, performance, fertility, udder health, primiparous cows

## Abstract

**Introduction:**

This article aimed to study cross-sectional associations between the performance of dairy farms and their corresponding culling proportions under the herd size constraint as imposed in 2018 by the new phosphate regulation in the Netherlands.

**Methods:**

To this end, production data from 10,540 Dutch dairy farms were analyzed to capture the inflow and outflow of both primiparous and multiparous cows. Farm performance was measured by 10 indicators structured in four areas of longevity, production, reproduction, and udder health. Farm culling proportions were represented by the overall culling (OC) and the number of culled primiparous cows in relation to (i) the total number of producing cows (PC), (ii) the number of producing primiparous cows (PPC), and (iii) the number of culled producing cows (POC). Spearman's rank correlation and weighted logistic regression were adopted to study associations.

**Results:**

In 2018, on average, 28% of producing cows were culled (OC). The number of primiparous cows culled represented 4.5% of the total number of producing cows (PC) and the mean proportion of culled primiparous cows was 18.8% of the total number of producing primiparous cows (PPC), and, of the total number of producing culled cows, 15% were primiparous cows (POC). However, the variance around the mean, and among individual farms, was high (SD 4–15% for all four culling proportions). Results from rank correlation showed very low-rank conformity (<12%) between the areas of production, reproduction, and udder health to the culling proportions. Results from logistic regression showed that higher farm levels of production and higher percentages of cows with poor udder health were associated with more overall culling but with less primiparous culling. For reproduction indicators, the associations were similar for overall and primiparous culling. However, except for the average age of culled animals, the odds ratios for indicators were close to 1 (range: 0.92–1.07 and 0.68–1.07 for OC and PPC, respectively), indicating only weak associations to culling proportions.

**Discussion:**

In conclusion, although the introduction of phosphate regulation resulted in an increased outflow of cattle, corresponding culling proportions were not associated with the level of farm performance measured in terms of production, reproduction, or udder health.

## 1. Introduction

Culling of dairy cows is one of the most complex aspects of dairy herd management. In accordance with their specific management styles, farmers follow different strategies in their decision-making process to cull dairy cows. Various studies have shown that the variation in the culling decision is not only related to individual cow performances and herd-level risk factors but also to factors such as farmers' behavior and management styles (1–4). Apart from these, changes in national or global policies regarding livestock production can alter the farmers' long-term strategies regarding the culling of dairy cows. A recent survival analysis of dairy cows in the Netherlands indicated that culling intensity may vary over the years due to changes in agricultural policy, while the reasons for the culling of individual cows remained the same (5).

In the current Dutch dairy production landscape, we see an increase in environmentally driven regulations, generally constraining the herd size (6). One example of such is the introduction of the phosphate regulation in 2018, which allows dairy farmers to produce phosphate from livestock manure only in accordance with the rights they have been granted. The number of phosphate rights granted to a dairy farmer was based on the number of cows kept in July 2015, subjected to a generic reduction of 8.3% (7). As a result, many farmers were forced to immediately reduce their livestock numbers, temporarily increasing the efflux of dairy cows (5) and youngstock. Nor et al. (3) and Haine et al. (1) found that in the Netherlands and Canada, the long-term culling rate of dairy farms was associated with herd-level factors such as the proportion of cows with elevated somatic cell count, herd average 305-day milk, and the herd average calving intervals. Results from Armengol and Fraile (8) suggested that variation between farm characteristics and the performance of herds could be important for culling differences between herds. However, these studies were aimed at the long-term associations between herd performance indicators and the magnitude of culling. There is a lack of literature on associations between herd performance and culling rate on dairy farms directly affected by policy-driven herd size constraints.

Previous research highlights that the cost of rearing a replacement heifer is not recovered until the second lactation (9); therefore, it is imperative that primiparous cows survive to their second lactation. Primiparous cows represent, as such, the potential by which the strategic performance goals set by the farmers' need to be achieved. With the introduction of a herd size restriction, such as with the phosphate regulation, youngstock and producing cows compete for the same production asset. Consequently, it is expected that primiparous cows, in particular, will not be culled upon the introduction of the phosphate rights system to give farmers some time to rebalance the ratio of youngstock needed for replacements to producing cows. Therefore, the culling of primiparous cows needed to be investigated when the phosphate rights system was introduced.

This study aimed to gain insights into the cross-sectional associations between annual performance indicators of Dutch dairy farms and their corresponding magnitudes of (i) overall culling and (ii) primiparous cow culling after the introduction of the herd size restricting phosphate regulation in the Netherlands. To study the associations between herd performance and intensified culling, 2018 production data from 10,540 Dutch dairy farms were used to capture the maximal policy influence on the inflow and outflow of both primiparous and multiparous cows.

## 2. Materials and methods

### 2.1. Data

The anonymized farm data used in this study were obtained from the Dutch cattle breeding company—Cattle Improvement Cooperative, CRV (CRV stands for “Coöperatie Rundvee Verbetering” in Dutch which is “Cattle Improvement Cooperative”). The data consisted of four datasets of farm-level records of Dutch dairy farms from the year 2018. These four datasets included herd composition data, production data, udder health data, and fertility data. In addition to these datasets, individual cow-level data of test-day MPR (MPR stands for Milk Production Registration which registers individual cow parameters at certain intervals) (10) from the year 2018 were obtained to evaluate the culling proportions of interest. Details of the individual datasets are presented in Supplementary Table 1. Overall, the data contained recordings on 14,609 Dutch dairy farms.

### 2.2. Data editing and variable selection

Active farms (active as in having at least four recordings in MPR data in 2018) were selected for analysis (*n* = 14,291 Dutch dairy farms). Farms that were not represented in all four farm-level datasets as well as farms with erroneous data records (for example, herd annual milk-fat percentage of 12%, etc.) were filtered out. Moreover, a commercial dairy farm was defined as a farm with 30 to 500 producing cows, hence excluding farms with less or more producing cows from further analysis. Farms with <30 cows are generally not commercial dairy farms, whereas farms with more than 500 cows are atypical in the Dutch dairy system (representing for instance, research farms). The final data included records of 10,540 farms (see Supplementary Table 2 for more detail on data editing steps).

The farm-level data on production and udder health were recorded at test-day intervals, whereas the herd composition and the fertility data consisted of annual recordings. The test-day records on production and udder health were converted to annual recordings by averaging over the number of test days in the MPR to make all four datasets reflect an annual scale. From the cow-level MPR data of the CRV, the number of primiparous cows culled was determined for all farms in the final dataset. These records, together with the total number of producing cows, total number of producing culled cows, and total number of primiparous cows (see Table 1; herd size variables), were used to calculate the following culling proportions for the year 2018:

(i) Overall culling (OC), the proportion of the total number of producing cows culled (n_culled) to the overall number of producing cows (n_tot) present in the herd of the farm in the year 2018, given by,

(1) $$\begin{array}{l} OC\;=\;\frac{n\text{\_}culled}{n\text{\_}tot} \end{array}$$

(ii) Primiparous culling (PC), the proportion of the number of 1st parity cows culled (primi_culled) to the overall number of producing cows present in the herd of the farm in the year 2018, given by,

(2) $$\begin{array}{l} PC\;=\;\frac{primi\text{\_}culled}{n\text{\_}tot} \end{array}$$

(iii) Primiparous–primiparous culling proportion (PPC), the proportion of the number of 1st parity cows culled (primi_culled) to the total number of 1st parity cows present in the herd (n_primi) of the farm, given by,

(3) $$\begin{array}{l} PPC\;=\frac{primi\text{\_}culled}{n\text{\_}primi} \end{array}$$

(iv)Primiparous-overall culling proportion (POC), the proportion of the number of 1st parity cows culled (primi_culled) to the total number of dairy cows culled, given by,

(4) $$\begin{array}{l} POC\;=\frac{primi\text{\_}culled}{n\text{\_}culled} \end{array}$$

**Table 1:** Description and summary statistics of culling proportions and farm-level performance indicators grouped by performance area of the evaluated dairy farms (*n* = 10,540).

	**Description**	**Min–max**	**IQR**	**Median**
**Culling proportions**				
OC^1^	Proportion of number of cows culled to overall number of producing cows	0.13–0.48	0.22–0.34	0.28
PC	Proportion of number of 1st parity cows culled to overall number of producing cows	0.0–0.41	0.02–0.07	0.04
PPC	Proportion of 1st parity cows culled to the number of 1st parity cows	0.00–1.00	0.10–0.27	0.18
POC	Proportion of 1st parity cows culled to total number of culled cows	0.00–0.83	0.09–0.23	0.15
**Longevity**				
Age_tot	Age of the dairy herd (days)	1,137–3,143	1,582–1,778	1,672
Age_culled	Age of culled dairy cows (days)	1,204–3,995	1,896–2,243	2,055
Life_prodn^a^	Lifetime production (kg)	5,379–41,365	19,604–24,898	22,183
**Production**				
avg_FPCM^a^, ^2^	Annual fat–protein corrected daily milk production (kg)	13.79–47.20	27.86–32.43	30.3
**Reproduction**				
Services_per_conception (nulliparous)^a^	Number of inseminations per calving for 0th parity cows (nulliparous)	0.50–7.00	1.35–1.85	1.6
Services_per_conception^a^	Number of inseminations per calving for3 1+ parity cows	0.00–5.50	1.52–2.12	1.8
AFC^a^	Age at 1st calving (days)	651.2–1266	748.3–803.5	772
Avg_DIM_first_service^a^	Interval in days in milk between last calving and first insemination (days)	41–490	79.27–103.91	89
Calv_int^a^	Calving interval (days)	353–781	392–420	404
**Udder health**				
Avg_high_SCC	Annual percentage of cows having high somatic cell count^3^ (%)	1.6–94.5	10.79–18.18	14.2
**Herd demographics**				
n_tot	Total number of producing cows in the farm in 2018	31–500	66–118	89
n_culled	Number of culled cows	5–228	17–35	25
n_primi	Total number of 1st parity cows	1–252	15–31	22
primi_culled^4^	Number of culled 1st parity cows	0–31	1.5–8.5	4

a Variables are farm averages calculated from the individual performance data of the producing cows on the farms from the data provided by the CRV.

1 OC, overall culling proportion; PC, primiparous culling proportion; PPC, primiparous-primiparous culling proportion; POC, primiparous-overall culling proportion.

2 Farm average milk yield on test-day converted to fat–protein corrected milk (FPCM) using the formula from Yan et al. (11): FPCM (kg) = (0.337 + 0.116 x fat % + 0.06 x protein %) x milk production (kg).

3 High somatic cell count is defined as cows having more than 150,000 cells/ml milk for primiparous and 250,000 cells/ml for multiparous cows.

4 Calculated from the cow-level MPR data obtained from the CRV (10).

From the available data, farm-level performance indicators were selected as a representative of four performance areas, namely, longevity, production, reproduction, and udder health. In any given performance area, when two variables were highly correlated (|r| > 0.75), the most relevant of the two was chosen. The final list of variables and their descriptions are shown in Table 1. Missing data were found in indicators of the reproduction area. Given the small proportion of missing to complete data, these records were excluded in all subsequent analyses except for descriptive statistics.

### 2.3. Descriptive statistics

The minimum-maximum, median, and interquartile range (IQR) of the performance indicators in four areas and the four culling proportions were calculated from the final data sample (Table 1). Similarly, the minimum-maximum, median, and IQR of herd demographics such as the number of producing cows, number of culled cows, and number of primiparous cows in the herd were calculated (Table 1).

To describe the culling proportions with respect to the herd size of the farms, farms were divided into six herd size groups of 31–50, 51–70, 71–90, 91–110, 111–150, and 151–500 producing cows. All four culling proportions (OC, PC, PPC, and POC) were plotted against the herd size groups by means of a boxplot.

### 2.4. Rank correlations

The performance indicators in all four areas were scaled and centered to the mean. Spearman's rank correlation test was performed to check for conformity in the ranking of farms based on these different performance indicators. In addition, rank correlation tests were performed between scaled indicators and the defined culling proportions (not scaled). The results of this procedure were interpreted as the degree of conformity between the ranking of farms based on performance indicators and the ranking based on culling proportions. Before the rank correlations, scatter plots of scaled indicators against culling proportions were drawn to investigate if any non-monotonic relationships exist.

### 2.5. Logistic regression model

To investigate the association of the (i) overall culling and the (ii) primiparous culling proportion to the performance indicators in a systematic format, two weighted logistic regression models were developed. In the first model, OC was the dependent variable. It was interpreted as the proportion of cows culled (binomial successes) to the total number of dairy cows (n-trials) with a binomial outcome. In the second model, PPC was selected as the dependent variable. PPC was interpreted as the proportion of primiparous cow culling (binomial successes) to total primiparous cows (n-trials) with a binomial outcome. The performance indicators shown in Table 1 were fitted in the model as associated independent variables. Due to a large amount of difference in the scales, the independent variables (performance indicators) were scaled and centered to the mean before fitting in both models. Herd effects were not included as random effects since only one annual record of each variable per farm was present. Post-modeling, the estimated effects were exponentiated to give the odds ratio per unit change in the scaled indicators. These effects were interpreted as the associations between the performance indicators and OC or PPC.

All the analyses and the data editing were performed in Rstudio with R 3.6.3 (12).

## 3. Results

### 3.1. Descriptive statistics

The mean herd size among the selected dairy farms was 105 producing cows with a median of 92 cows (Table 1). Over the year 2018, 28% of producing cows were culled on average (OC). The number of primiparous cows culled represented 4.5% of the total number of producing cows (PC). The mean proportion of culled primiparous cows was 18.8% with respect to the total number of producing primiparous cows (PPC), and of the total number of culled cows, 15% were primiparous cows (POC). The average herd longevity (Age_tot) was 1,688 days (~4 years, 7 months), whereas the average age of culled cows (Age_culled) was 2,089 days (~5 years, 9 months).

**Figure 1:**
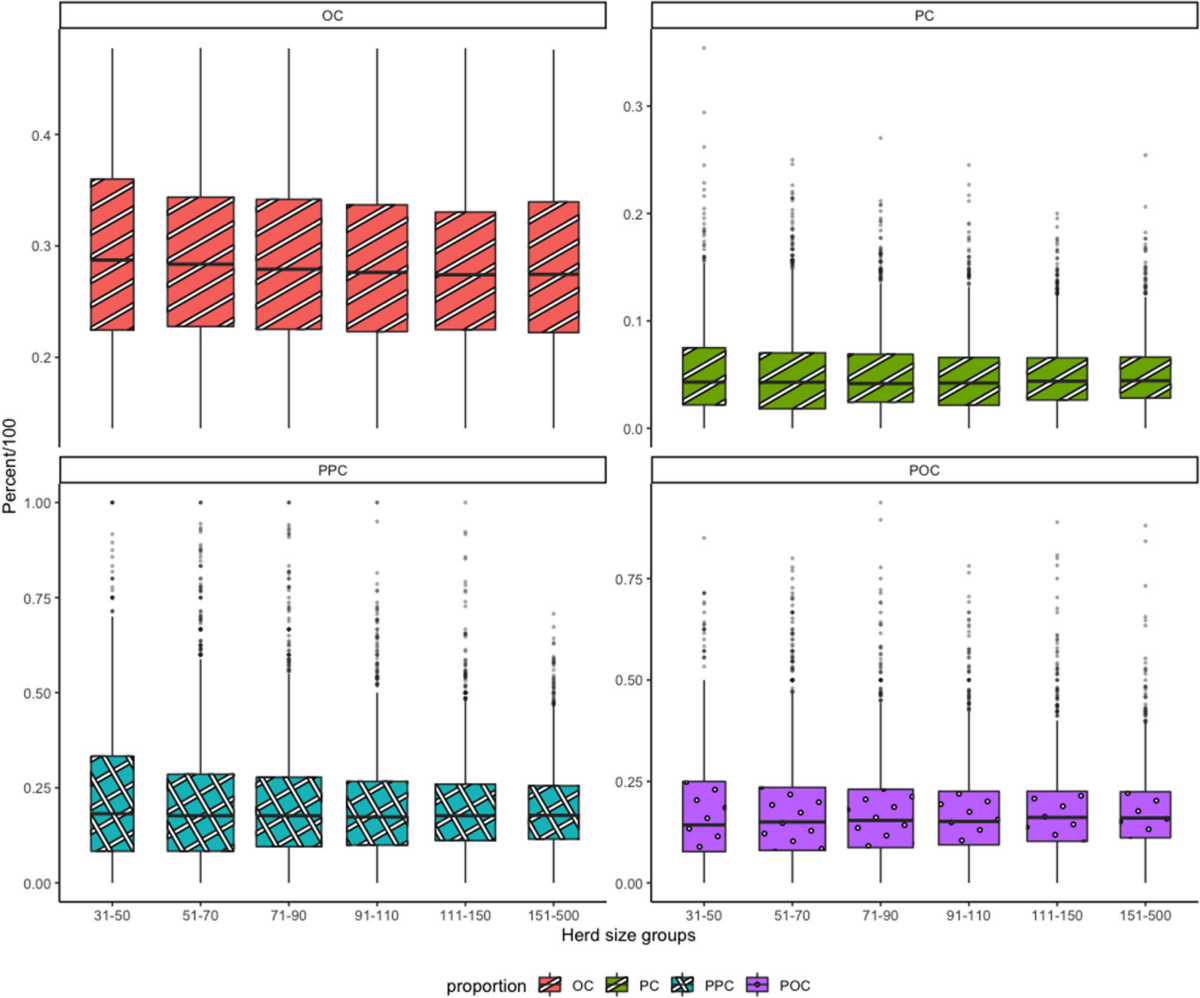
Distribution of culling proportions across herd size groups.^.^ OC, overall culling proportion; PC, primiparous culling proportion; PPC, primiparous-primiparous culling proportion; POC, primiparous-overall culling proportion. Herd size groups (x-axis) represent the number of producing cows in the farms in 2018.

Figure 1 shows the variation in the evaluated culling proportions per herd size group. In general, the means of four culling proportions were similar between all the herd size groups. The variables PPC and POC were almost equal in means among the groups but the variation around the mean was different. The variation in all four proportions was higher for the smaller herd size groups and smaller for larger herd size groups. The smallest variation in all four proportions was in the 151–500 producing cow group. It was also seen that there was high variation within each herd size group. From Figure 1, the mean of PC was considerably lower than that of OC for all the herd size groups. Moreover, the overall mean of POC, which was the proportion of primiparous cows culled to total culled cows, was 16%. This showed that the primiparous cows were a minority in the group of cows that were culled in 2018.

### 3.2. Rank correlations

**Figure 2:**
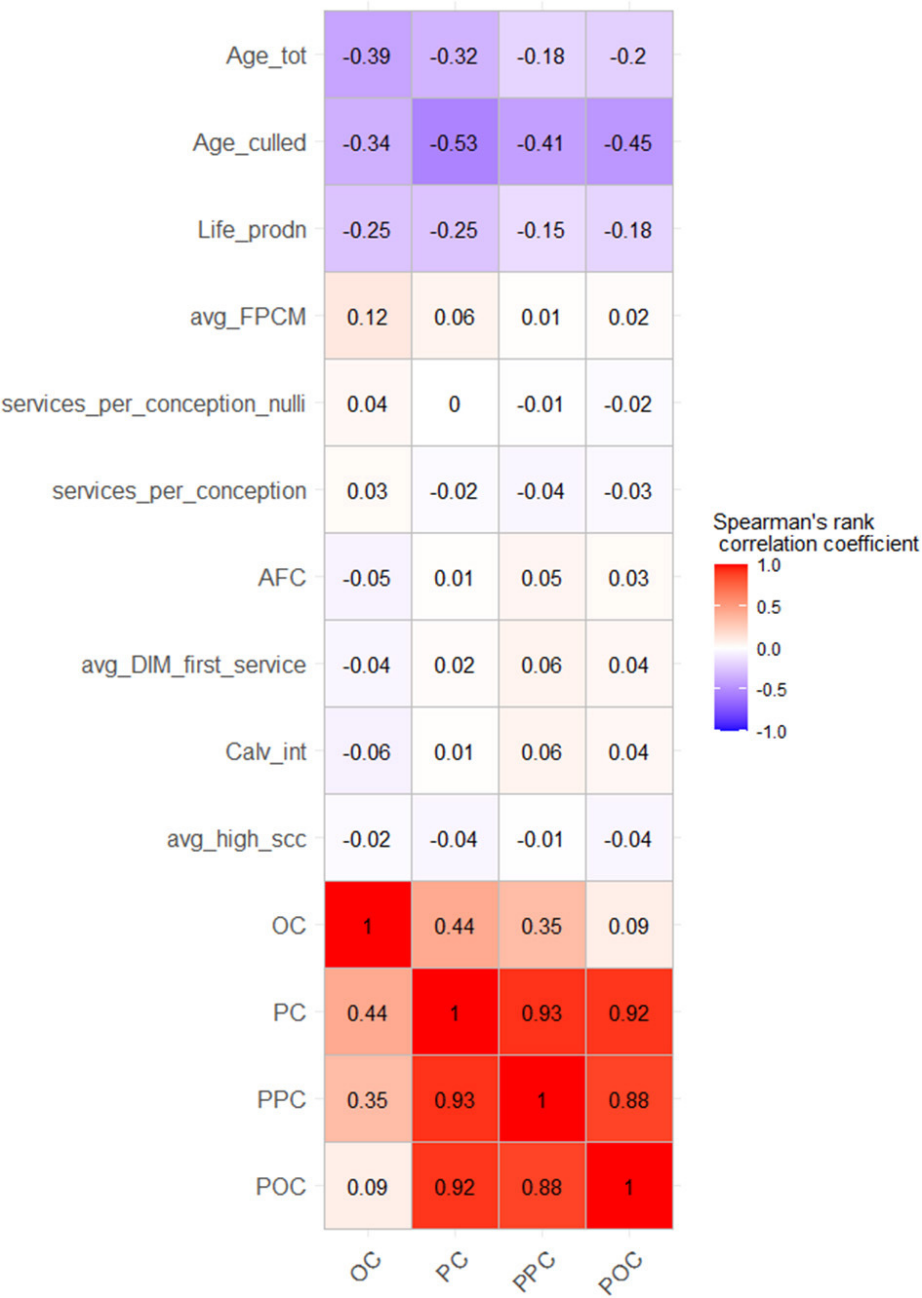
Spearman's rank-correlation matrix of correlations between the four culling proportions and performance indicators. Age_tot, total herd average age; Age_culled, herd average age of culled cows; Life_prodn, lifetime production of milk; Avg_FPCM, herd average daily fat-protein corrected milk; services_per_conception_nulli, mean number of inseminations per calf (in nulliparous cows); services_per_conception, mean number of inseminations per calf (in multiparous cows); AFC, age at first calving; avg_DIM_first_service, interval of days in milk (DIM) between last calving and first insemination; Calv_int, herd average calving interval; avg_high_scc, proportion of cows in herd with high somatic cell count; OC, overall culling proportion; PC, primiparous culling proportion; PPC, primiparous-primiparous culling proportion; POC, primiparous-overall culling proportion.

Spearman's rank correlation coefficients of scaled performance indicators and the culling proportions were calculated. From Figure 2, longevity indicators of herd average age and lifetime milk production had higher correlation coefficients of −0.39 and −0.25 with overall culling proportion (OC) compared with the three primiparous culled cow proportions (PC, PPC, and POC). Similarly, the average FPCM had a slightly higher rank correlation of 0.12 with OC compared with the primiparous culled cow proportions. In the reproduction area, services per conception for nulliparous and multiparous cows, age at first calving, and calving interval had opposite but very weak correlations with OC compared with PC, PPC, and POC.

There was a high-rank correlation (> 0.8) between the primiparous culled cow proportions, namely, PC, PPC, and POC. The rank correlation between the overall culling proportion (OC) and PPC, PC, and POC was 0.35, 0.44, and 0.09, respectively. From Figure 2, rank correlations between all longevity indicators and all four culling proportions were moderately high to low (range: −0.53 to −0.15), indicating a different approach to primiparous and multiparous cow culling. For all other performance indicators, the rank correlations with culling proportions were very low (rho <0.2; Figure 2). This showed that there was very little rank conformity between the performance areas (except the longevity area) and primiparous culling proportions.

Scatter plots drawn between scaled performance indicators and culling proportions did not show any non-monotonic relationship between the indicators and proportions (Supplementary Figures 1A–D).

**Figure 3:**
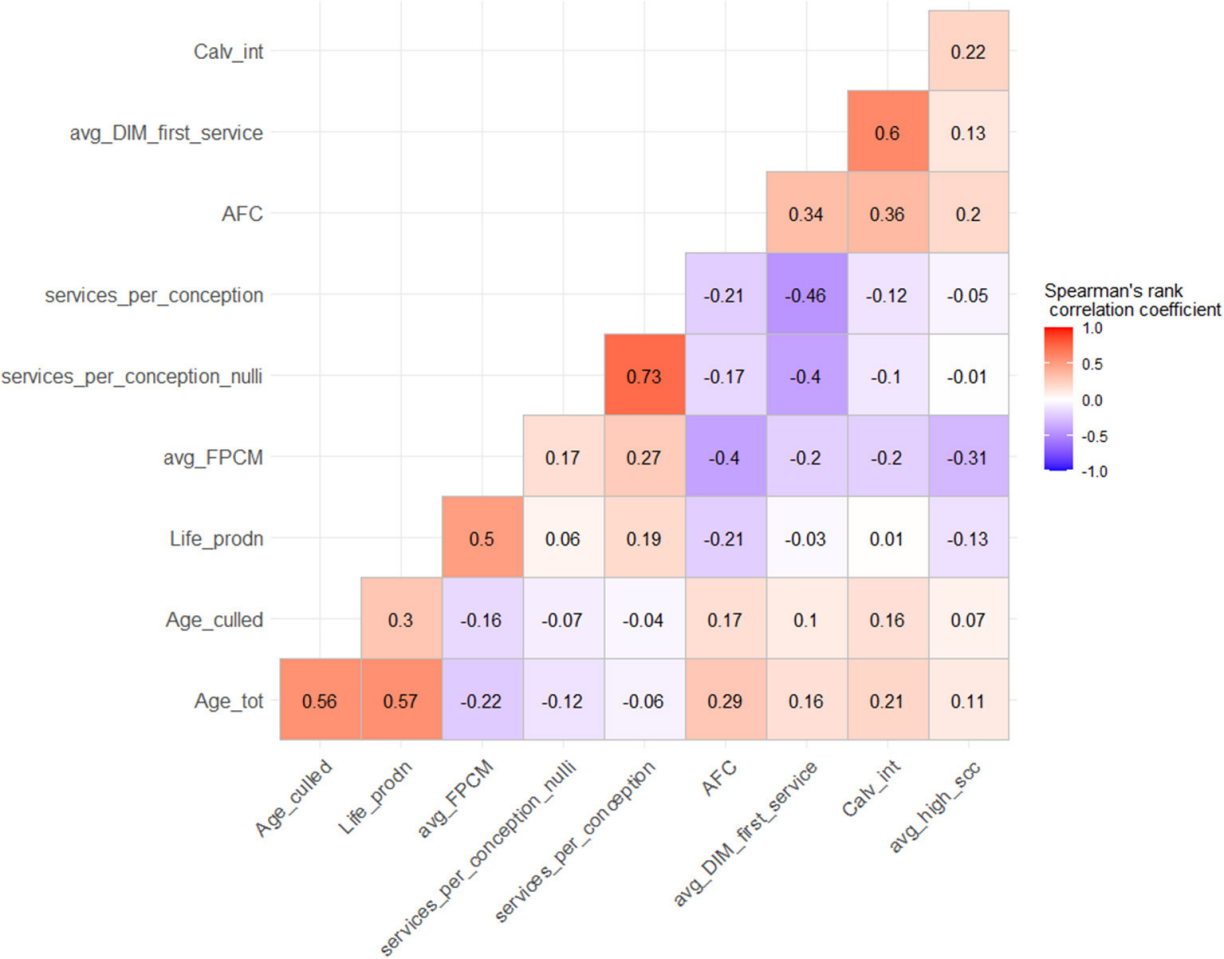
Spearman's rank-correlation matrix (lower triangle) of performance indicators. The variables in rows and columns are the 10 performance indicators in four areas. Age_tot, total herd average age; Age_culled, herd average age of culled cows; Life_prodn, lifetime production of milk; Avg_FPCM, herd average daily fat-protein corrected milk; services_per_conception_nulli, mean number of inseminations per calf (in nulliparous cows); services_per_conception, mean number of inseminations per calf (in multiparous cows); AFC, age at first calving; avg_DIM_first_service, interval of days in milk (DIM) between last calving and first insemination; Calv_int, herd average calving interval; avg_high_scc, proportion of cows in herd with high somatic cell count. Diagonal (self-correlations) not shown.

From Figure 3, the rank correlations between the indicators belonging to the longevity and reproduction areas ranged between 0.3 to 0.57 and −0.46 to 0.73, respectively. Rank correlations higher than 0.75 were not present, as these were used as threshold settings in the variable selection. The areas of production and udder health had only one indicator each. The rank correlations between indicators belonging to different performance areas were generally low, as indicated by the range varying from an absolute minimum of 0.01 (rho = 0.01) between calving interval and lifetime milk production to an absolute maximum of 0.5 (rho = 0.5) between herd average lifetime production and herd average FPCM yield (Figure 3).

### 3.3. Logistic regression model

In the weighted logistic regression model, the associations between the performance indicators and the culling proportions of OC and PPC were tested by odds ratio and the results are shown in Table 2. An odds ratio of more than 1 is associated with a higher culling proportion, whereas an OR < 1 is associated with a lower culling proportion.

**Table 2:** Summary of results: multivariable fractional logistic regression models with overall culling (OC) or primiparous–primiparous culling proportion (PPC) as dependent variable against scaled herd performance indicators in four areas.

	**OC**		**PPC**	
**Indicator** ^a^	**OR (95% CI)**^b^, ^c^	***p-*** **value**	**OR (95% CI)**^b^, ^c^	***p-*** **value**
Intercept	0.39 (0.38–0.39)	< 0.001	0.23 (0.22–0.23)	< 0.001
**Reproduction** ^b^				
Services per conception (nulliparous)	1.01 (0.99–1.02)	0.81	0.99 (0.98–1.01)	0.69
Services per conception (multiparous)	1.01 (0.99–1.02)	0.20	0.99 (0.97–1.01)	0.28
Average DIM at first service	1.00 (0.99–1.00)	0.60	1.03 (1.01–1.04)	< 0.001
Calv_int	1.02 (1.01–1.02)	< 0.001	1.12 (1.10–1.14)	< 0.001
AFC	1.01 (1.00–1.02)	< 0.001	1.01 (0.99–1.02)	0.08
**Longevity** ^b^				
Age_tot	0.96 (0.95–0.96)	< 0.001	1.11 (1.10–1.13)	< 0.001
Age_culled	0.92 (0.92–0.93)	< 0.001	0.63 (0.62–0.64)	< 0.001
Life_prodn	0.92 (0.91–0.93)	< 0.001	0.95 (0.93–0.97)	< 0.001
**Production** ^b^				
avg_FPCM	1.08 (1.07–1.08)	< 0.001	0.99 (0.97–1.01)	0.50
**Udder health** ^b^				
avg_high_SCC	1.01 (1.00–1.01)	< 0.001	0.97 (0.96–0.98)	< 0.001

a All indicators were scaled (centered to mean) due to differences in scale.

b OR, odds ratios; services per conception, number of inseminations/services per calving; average DIM at first service, interval in days in milk (DIM) between last calving and first insemination; AFC, age at first calving; Age_tot, total herd average age; Age_culled, herd average age of culled cows; Life_prodn, lifetime production of milk; Avg_FPCM, average fat-protein corrected milk; Avg_high_SCC, average percentage of high SCC cows in the herd.

c All values rounded to two digits after the decimal.

In the OC model, higher herd averages of the calving interval, FPCM, and proportion of cows with high SCC were associated with higher overall culling from reproduction, production, and udder health areas, respectively. Three of the four longevity indicators, namely, higher herd average age of cows, higher average age of culled cows, and higher herd average lifetime production were associated with less overall culling (Table 2). In the PPC model, longer intervals between the last calving to first insemination and longer calving interval were both associated with higher primiparous culling from the reproduction area. Moreover, in the PPC model, unlike in the OC model, the production indicator and the udder health indicator were negatively associated with primiparous culling risk. In the PPC model (Table 2), one longevity indicator, higher age of culled animals, was associated with less primiparous culling (OR = 0.69), whereas higher herd average age of cows and higher herd average age of culled cows and age at first calving were associated with higher primiparous culling proportion.

## 4. Discussion

This study aimed to gain insights into the cross-sectional associations between annual performance indicators of Dutch dairy farms and their corresponding magnitudes of (i) overall culling and (ii) primiparous cow culling under the herd size restriction induced by the introduction of phosphate regulation in the Netherlands. The number of phosphate rights granted to a dairy farmer was based on the number of cows kept in 2015, subjected to a generic reduction of 8.3% (7). As most dairy herds expanded after the abolishment of the milk quota in 2015, where the average dairy herd size increased from 85 in 2014 to 97 producing cows in 2016 (13), it was expected that most dairy farmers had to adjust their culling magnitude in response to the new policy.

The results indicated an overall culling rate (OC) of 28% (SD 8%), which was only slightly different than the OC in the previous years 2015, 2016, and 2017 of 22% (SD 7%), 24% (SD 8%), and 30% (SD 8%), respectively (Unpublished data; OC calculated on the same sample size of 10,540 herds). Dairy farmers could reduce herd sizes by culling dairy cows without replacement and/or by increasing the outflow of youngstock. According to official census data (Central Bureau of Statistics), the total number of dairy cows in the Netherlands reduced between April 2017 and April 2018 by 4% to 1.62 million dairy cows (14, 15). During the same period, the number of youngstock, however, decreased by 14% to 1.03 million cows (14). This indicates that in the year of the policy introduction, farmers responded initially by adjusting the herd size of their youngstock, explaining the moderate increase in OR.

Only 16% (SD 9%) of the culled cows were primiparous (POC) which is comparable to the 17.5% measured in the years 2007–2012 (10). This indicated that primiparous cows formed a minor proportion of the overall culling magnitude effected by the farmers on their herds. This was in line with the earlier findings of Archer et al. (9). From Figure 1, it can be seen that there was a large variation around the mean for all four culling proportions (OC, PC, PPC, and POC) in all herd sizes, indicating that farmers varied in their culling strategies and that there was no indication of a uniform response with respect to the policy changes.

Unfortunately, we did not have access to the data regarding the culling of primiparous cows in the years 2016 and 2017. Moreover, the available data on performance indicators were in the form of annual summaries either on the cow or farm level and not on a monthly or quarterly basis. Therefore, it was not possible in this study to compare or track changes in farm performances or the culling rates for primiparous cows before and after the application of the phosphate rights system in the Netherlands. Rather, this study focused on the immediate associations between the overall and primiparous cow culling and the performance of farms after the policy changed. Further study representing changes or alterations in the culling rate before and after the application of the phosphate rights system is required to completely assess the effect of the new policy on dairy farm management in the Netherlands.

The rank conformity between production, reproduction, and udder health indicators and the four culling proportions was weak to non-existent (Figure 2), indicating that the variation in culling magnitude was not associated with the annual herd performance. From Table 2, reproduction, production, and udder health indicators were found to be significantly associated with primiparous and overall culling (OC and PPC), in contrast to the rank correlation findings. Particularly, production and udder health indicators had opposite associations (positive for OC and negative for PPC) to the culling proportions. This was in line with the findings of Oltenacu et al. (16), who found that primiparous cows were at higher risk of culling due to health problems compared with older cows. Nevertheless, based on the odds ratios, all significant associations ranged between weak and moderate at best. This indicated that the extent of primiparous and overall culling varied irrespective of farm performance. Based on this, we theorized that the variation in culling was not driven by farm performance level.

In all the statistical analyses, only longevity indicators were consistently found to be associated with the culling proportions. However, these associations can be explained numerically (not causally) since there is a direct functional relationship between current longevity and previous culling (17). For example, the indicators such as herd-average age of culled animals (Age_culled) and herd average age of cows (Age_tot) were directly influenced by the proportion of young animals such as primiparous cows being culled on the farm in previous years. On the other hand, these associations may be suggestive of differences in the management and behavior of farmers. For example, some farmers give more chances to primiparous cows, and culling for performance goals is focused on 2nd parity cows when policy changes are applied. Whereas some farmers may judge 1st parity cows more critically, leading to premature culling and so on to reduce herd sizes. This is irrespective of which strategy is best for maximum overall performance. Therefore, it was not possible to provide a straightforward interpretation of the evaluated rank conformity between the longevity area and the primiparous cow culling proportions.

It can be argued that the effects of policy changes such as the phosphate regulation affect farm performance in the medium to long term instead of the short term. Especially when considering the difference in the relative decrease in youngstock compared with dairy cows, disturbing the influx–efflux balance on a farm (5). Longitudinal data on longer term effects were, however, not available during this study. Hence, targeting mid- to long-term associations was beyond the scope of the study but is certainly very interesting. The lack of longitudinal data might also explain the weak relationships found between the performance areas and culling proportions compared with the results of long-term studies such as that by Nor et al. (3).

Figure 3 indicated that there was no monotonic relationship between the performance indicators from different areas. This finding agrees with the insights obtained from the factor analyses on longitudinal data of Haine et al. (1) and the findings of Brotzman et al. (18) who used a combination of principal component and cluster analyses. It seems that farms are not ranked high or low consistently among the different areas, and integrative approaches such as factor analyses would not solve the underlying issue. There seems to be a gap in approaches or methods to describe overall farm performance in many areas independently.

## 5. Conclusion

In conclusion, there was a rise in overall culling on Dutch dairy farms during the year 2018 after the introduction of the phosphate rights system in national agricultural policy. Moreover, there was a high degree of variation between the culling rates of Dutch dairy farms around the national mean. Primiparous cow culling formed a minor proportion of the overall culling rates of the farms, indicating that young producing cows were not targeted by farmers for altering herd size post-policy change in the year of study. However, overall primiparous cow culling was not systematically related to the performance level of dairy farms in reproduction, production, and udder health areas in the same year.

## Data availability statement

The data analyzed in this study is subject to the following licenses/restrictions: authors do not have the authorization to share the data. Models and secondary data generated during analyses that are part of this article will be made available on Github. Requests to access these datasets should be directed to PK, pranav.kulkarni@wur.nl.

## Author contributions

PK, MM, MN, and WS conceived and designed the study and framework. PK carried out the data cleaning, performed the calculations, analyses, and wrote the manuscript with input from all authors. MM and MN provided daily supervision. WS oversaw overall direction and planning. All authors contributed to the article and approved the submitted version.
