# Supplementary material for: Associations between dairy farm performance indicators and culling rates under policy-driven herd size constraints
Source: Front Vet Sci. 2023 Mar 23;10:1062891. doi: 10.3389/fvets.2023.1062891 (PMC10076624; doi:10.3389/fvets.2023.1062891)
Supplement: Supplementary file 1 [file Data_Sheet_1.docx]

Supplementary Material

# Supplementary tables

Supplementary Table 1. Details of CRV^a^ datasets used in this study

| Name of dataset ^a^ | Description | Variables ^1^ | Recording moment |
| --- | --- | --- | --- |
| Herd composition data | Annual figures of herd averages and number of animals on farms | UBN ^2^, Age_tot, Age_culled, Life_prodn, n_tot, n_culled, n_primi, Calv_Int | Annual, farm level |
| Fertility data | Annual figures of reproduction and fertility related indicators | UBN ^2^, Primi/Multi avg_DIM_first_service, AFC, services per conception | Annual, farm level |
| Udder health data | Test-day farm level figures of udder health | UBN ^2^, test_date, test-day SCC, %high_SCC, %new_inf | Test-day, farm level |
| Production data | Test-day farm level recordings of milk yield related indicators | UBN ^2^, test_date, test-day milk, test-day fat, test-day protein, 305-day milk, 305-day fat, 305-day protein, BSK, net value | Test-day, farm level |
| Milk Production registration | Test-day cow level records on milk production of cows | Animal Identifier, UBN^2^, test_date, parity, test-day milk, Lactation Value, etc. | Test-day, cow level |

^a^ Names of datasets translated into English from original Dutch names

^1^ Detailed description and full names of relevant variables in Table 1 of manuscript

^2^ UBN is farm identifier number anonymized at source by CRV

Supplementary Table 2. Steps of data selection and editing performed to CRV farm-level data of 2018

| Step | Process | Number of farms |
| --- | --- | --- |
| 0 | Raw data from CRV:   - 4 data sets representing farm-level records 1 data set representing MPR records on cow level | 14,609 |
| 1 | Exclude farms with < 4 MPR recording moments | 14,291 |
| 2 | Exclude farms with < 30 or >= 500 producing cows | 12,920 |
| 3 | 1. Calculate annual averages of test-day records on production and udder health 2. Exclude farms with erroneous and unrealistic values (eg. Annual Farm milk-fat yield of 12%, etc.) 3. Include only farms that are represented in all 4 herd level datasets | 12,652 |
| 4 | 1. Calculate culling proportion variables OC, PC, PPC, POC ^1^ 2. Include only farms with OC value between 5-95% interval (90 percentile) | 10,540 |

^1^ Abbreviations OC: proportion of number of cows culled to overall number of producing cows in the Farm, PC: proportion of number of 1^st^ parity cows culled to overall number of producing cows, PPC: proportion of 1^st^ parity cows culled to the number of 1^st^ parity cows producing in the Farm, POC: proportion of 1^st^ parity cows culled to total number of culled cows

^1^ Refer to Eqn 1 to 4 in main body text for formulae

# Supplementary figures


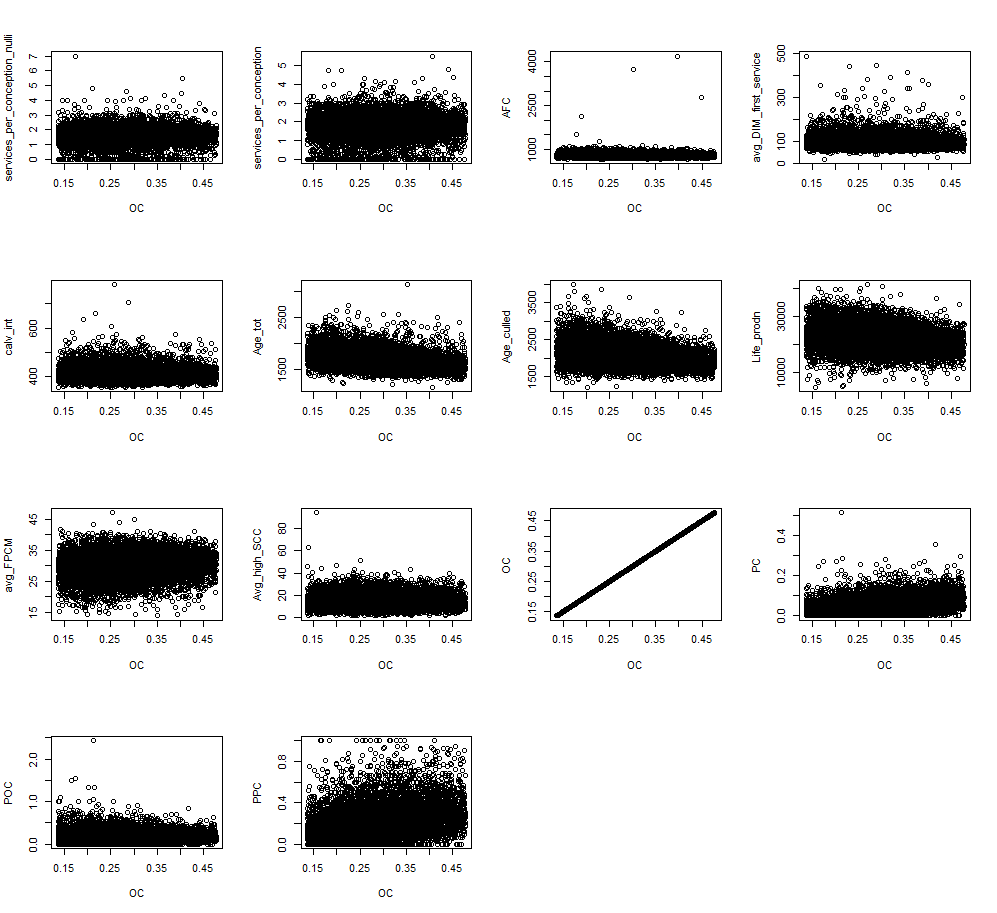


Supplementary figure 1 A. Scatter plots of performance indicators (y-axis) against overall culling proportion OC (x-axis)


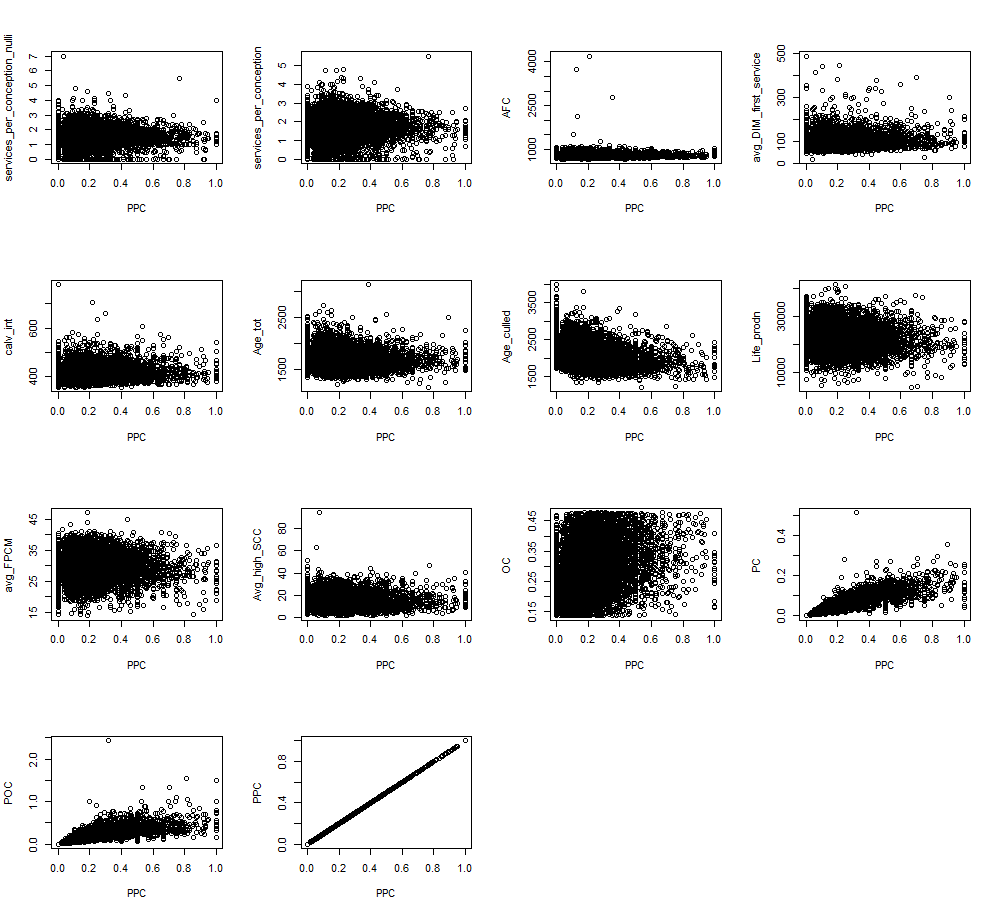


Supplementary figure 1 B. Scatter plots of performance indicators (y-axis) against Primiparous-primiparous culling proportion PPC (x-axis)


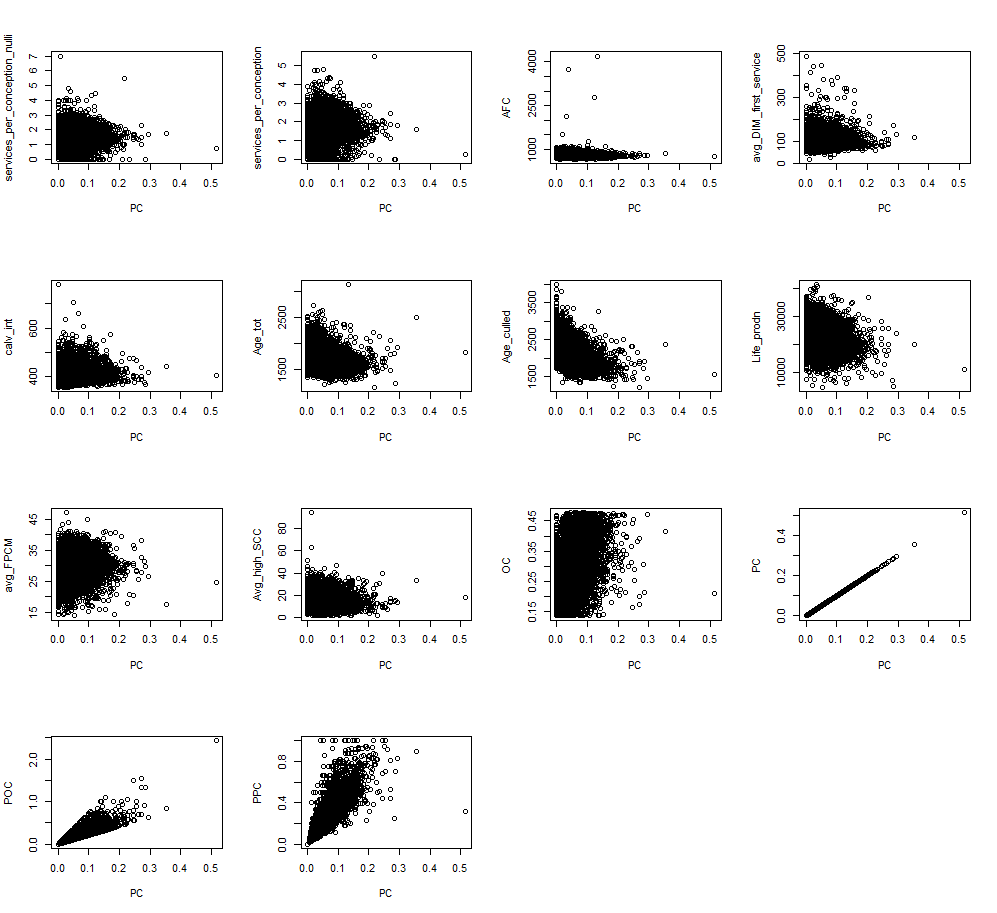


Supplementary figure 1 C. Scatter plots of performance indicators (y-axis) against Primiparous culling PC (x-axis)


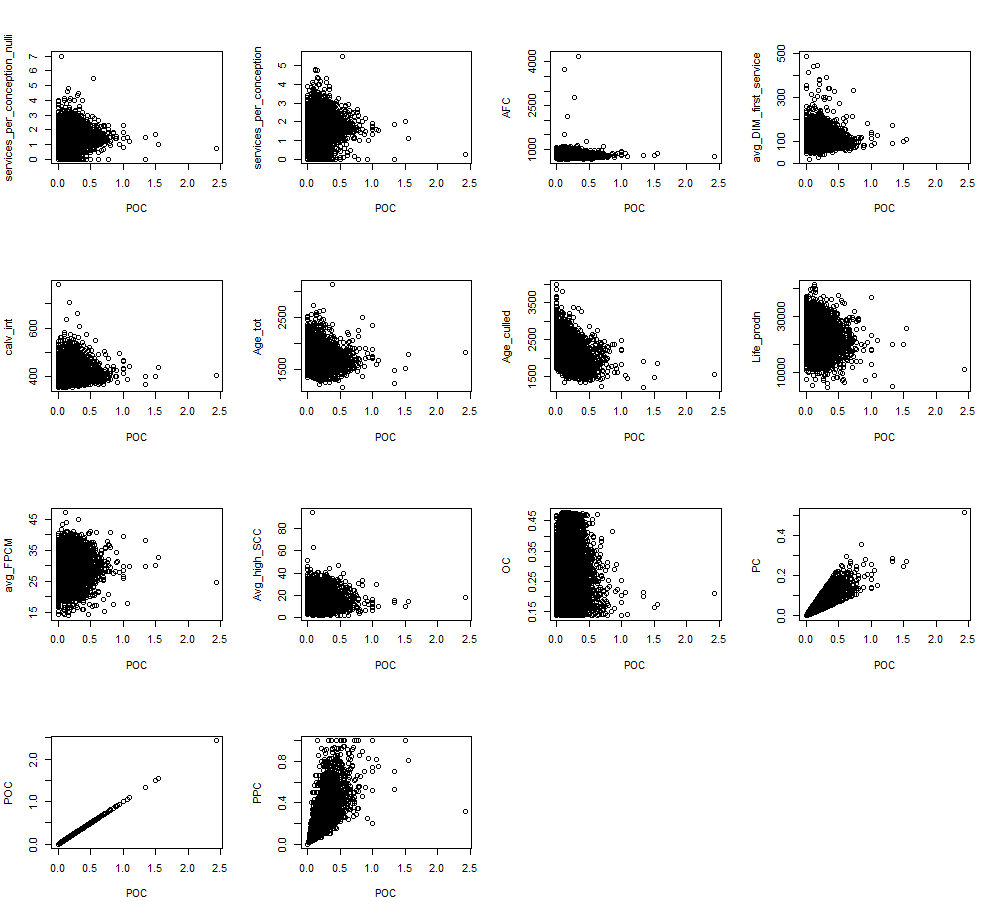


Supplementary figure 1 D. Scatter plots of performance indicators (y-axis) against Primiparous-overall culling POC (x-axis)
